# Determinants of Early Response to Low-Intensity Extracorporeal Shockwaves for the Treatment of Vasculogenic Erectile Dysfunction: An Open-Label, Prospective Study

**DOI:** 10.3390/jcm8071017

**Published:** 2019-07-11

**Authors:** Roberto Vita, Salvatore Benvenga, Bruno Giammusso, Sandro La Vignera

**Affiliations:** 1 Endocrinology, Department of Clinical and Experimental Medicine, University of Messina, 98125 Messina, Italy; 2 Master Program on Childhood, Adolescent and Women’s Endocrine Health, University of Messina, 98125 Messina, Italy; 3 Interdepartmental Program of Molecular & Clinical Endocrinology, and Women’s Endocrine Health, University Hospital, Policlinico Universitario G. Martino, 98125 Messina, Italy; 4 Andrology Unit, Policlinico Morgagni, 95125 Catania, Italy; 5 Department of Clinical and Experimental Medicine, Policlinico “G. Rodolico,” University of Catania, 95123 Catania, Italy

**Keywords:** extracorporeal shockwave therapy, low intensity, erectile dysfunction, impotence, penis

## Abstract

The aim of this study was to expand existing literature on the effects of cardiovascular risk factors on the outcome of low-intensity extracorporeal shockwaves therapy (LIESWT), and to evaluate the role of hormone concentrations. Twenty patients with long-standing, PDE5i-resistant, vasculogenic erectile dysfunction (VED) were treated with six weekly sessions of LIESWT (9000 pulses). After a three-week break, four poor responders underwent another six weekly sessions. Rigidity score (RS) questionnaire was administered at baseline (T0), last session (T1), and three months after LIESWT (T2), while the Improvement component of the Clinical Global Impression of Change (CGIC-I) and the International Index of Erectile Function-5 (IIEF-5) questionnaires were administered at T1 and T2, and at T0 and T2, respectively. At T0 serum luteinizing hormone (LH), testosterone, sex hormone binding globulin (SHBG), calculated free testosterone, and prolactin levels were also recorded. At T1 and T2, 12/20 (60%) and 11/20 (55%) patients reached a RS ≥ 3; 16/20 (80%) and 13/20 (65%) improved their erections variably. Testosterone levels correlated positively with CGIC-I at T1. Patients < 65 years and those nonhypercholesterolemic had higher RS at T1 and T2. Age correlated negatively with RS at T1 and T2. At T0, diabetic patients had lower IIEF-5 scores, but those with RS ≥ 3 at T1 had higher IIEF-5 compared to those with RS < 3. Also, diabetes duration correlated inversely with IIEF-5 at T0. At T2, IIEF-5 improved significantly by an average of 2.8-points. We confirm safety and effectiveness of LIESWT for the treatment of VED. Age ≥ 65 years, diabetes, and hypercholesterolemia influence early and negatively the outcome of LIESWT.

## 1. Introduction

Erectile dysfunction is the inability to attain and maintain a penile erection hard enough for satisfactory sexual intercourse lasting at least 6 months [1]. The prevalence of erectile dysfunction increases with age and is higher in patients with certain systemic diseases, such as diabetes mellitus, hypercholesterolemia, and hypertension [1]. Other than resulting in reduced self-esteem and quality of life, there is evidence that erectile dysfunction is a marker of cardiovascular disease, because it is independently associated with stroke, coronary heart disease, and all-cause mortality [2,3,4,5,6,7].

One of the most frequent causes of erectile dysfunction is penile arterial insufficiency (vasculogenic erectile dysfunction), which can be caused by hypertension, diabetes mellitus, hyperlipidemia, and cigarette smoking, all of them having endothelial dysfunction as a common denominator [1]. Up to 40% of patients with vasculogenic erectile dysfunction do not respond or respond poorly to phosphodiesterase type 5 inhibitors (PDE5i) even at their maximum dose [8]. Furthermore, a number of men do not tolerate the bothersome side effects of PDE5i, rendering them drugs that are considered high-dropout-rate drugs [9]. 

Among alternative treatments for vasculogenic impotence, low-intensity extracorporeal shockwaves therapy (LIESWT) stood out in the recent years [1,10]. Shockwaves are longitudinal acoustic waves that are transmitted through tissues and have been successfully used in different settings. LIESWT is thought to be capable to improve penile perfusion by inducing neo-vascularization [11,12]. Vardi et al. showed for the first time the efficacy of LIESWT for the treatment of vasculogenic erectile dysfunction. Improvement in the erectile domain of IIEF-5 was evident already at one month after treatment, and it was maintained at three and six months [12]. Subsequently, the same group carried out a randomized, double-blind, sham-controlled study, in which IIEF-5 score improved significantly after one month of LIESWT compared to patients who received sham treatment [10]. It is noteworthy to notice that all the recruited patients were PDE5i responders who had withdrawn PDE5i ingestion for one month prior to commencement of the study [10]. Interestingly, in a subsequent double-blind, sham-controlled study, the same authors showed a ~50% response rate in PDE5i nonresponders [13]. 

The first systematic review by Fojecki et al. showed inconsistent results of LIESWT on the IIEF score, whereas improvement in the rigidity score (RS) score implied that PDE5i responders might recover natural erections after LIESWT [14]. Subsequently, Lu et al. reviewed 14 studies on vasculogenic and neurogenic erectile dysfunction, alone or in combination with either Peyronie’s disease or chronic pelvic pain [15]. They found that LIESWT was capable of improving IIEF and RS at three months and one month after treatment, respectively [15]. A recent meta-analysis of seven randomized-controlled trials including over 600 patients with vasculogenic erectile dysfunction showed that LIESWT improves significantly erections compared with sham therapy [16]. The authors found also high heterogeneity between studies [16]. Indeed, differences in LIESWT devices (linear vs. radial vs. focused, electohydraulic vs. electromagnetic vs. piezoelectric vs. piezomagnetic), treatment protocols (number of pulses per session, number and frequency of sessions, medium for shockwaves transmission, sites of application of the probe), and inclusion criteria exist, limiting generalization of these findings [11,17]. Particularly, one of the most important differences between LIESWT devices consists in the way shockwaves are delivered: Whereas shockwaves are focused at a predetermined tissue depth by focused devices, a larger area is covered by shockwaves generated by linear devices. Radial shockwaves have been also proposed for the treatment of erectile dysfunction, as they can generate a pressure wave that propagates as a spherical wave [18]. Indeed, literature on focused shockwaves is more robust, whereas radial shockwaves are not shockwaves *sensu stricto* [19,20]. 

So far, only three studies have evaluated the impact of cardiovascular risk factors on the outcome of LIESWT [21,22,23], showing the negative effect of age [21] and diabetes mellitus [22], but with contrasting results for hypertension and dyslipidemia [21,22,23]. To expand existing literature on this topic, and to evaluate the effect of hormone concentrations, we performed an open-label prospective study.

## 2. Materials and Methods

We carried out an open-label, single-arm, prospective study by selecting 20 patients with long-standing (≥2 years) erectile dysfunction. Inclusion criteria were: (i) Having a vasculogenic erectile dysfunction; (ii) being either a nonresponder or a poor-responder to at least one PDE5i; (iii) giving informed written consent. Cavernosal artery insufficiency was diagnosed by a peak systolic velocity lower than 30 cm/s upon stimulation with intracavernosal 10 μg alprostadil. Exclusion criteria were: (i) Pure psychogenic erectile dysfunction; (ii) neurogenic erectile dysfunction; (iii) pelvic surgery; (iv) hypogonadism; (v) hypothyroidism/hyperthyroidism; (vi) hyperprolactinemia; (vii) recovery from any cancer within the past 5 years.

Shockwaves were delivered by an electrohydraulic unit with a focused shockwave source (Omnispec ED1000, Medispec Ltd, Gaithersburg, MD, USA). The attached probe was directed at the penis and the crura upon applying commercial gel for ultrasonography. During a 20-min session, a total of 1500 pulses were delivered at a frequency of 120 pulses per minute, and with an energy density of 0.09 mJ/mm^2^. Upon stretching the penis manually, 300 pulses were delivered at distal, mid, and proximal shaft. Subsequently, another 300 pulses were delivered at right and left crura. The protocol consisted of 6 weekly sessions. After a 3-week break, four nonresponders/poor responders (i.e., those who did not experience any improvement in penile hardness) underwent another 6 weekly sessions, for a total of 12 sessions. During LIESWT cycle(s), we advised the patients to maintain their regular sexual activity and their erectogenic therapy, that is PDE5i (all patients), intracavernosal alprostadil (1 patient), or both (1 patient).

Erectile dysfunction was evaluated by three patient-reported questionnaires: (i) The International Index of Erectile Function-5 (IIEF-5), which was administered at the first visit, namely a few days prior to the commencement of LIESWT (baseline, T0); (ii) RS, which was administered at T0 and at the last LIESWT session (T1), namely the sixth for patients who underwent 6 sessions, or the sixth and the twelfth for patients who underwent 12 sessions (see above); (iii) the Improvement component of the Clinical Global Impression of Change (CGIC-I), which was administered at T1. IIEF-5, RS, and CGIC-I were also administered 3 months after completion of LIESWT sessions (T2). IIEF-5, also known as Sexual Health Inventory for Males, is the abridged, 5-item version of the 15-item IIEF, and assesses two domains, namely the erectile function (questions 2, 3, and 4) and the intercourse satisfaction (questions 1 and 5) [24]. Severe, moderate, mild to moderate, and mild erectile dysfunction are identified by an IIEF-5 score of 1–7, 8–11, 12–16, and 17–21, respectively [24]. RS, also known as Erection Hardness Score, is a single-item, 5-point scale that evaluates penile hardness and the ability to have an intercourse. RS ranges from 0 (no erection) to 4 (complete erection) [25]. CGIC-I is a single-item, 7-point scale that rates changes in erectile function compared with initiation of the treatment. CGIC-I ranges from −1 (very much worsened) to +3 (very much improved) [26].

Furthermore, the revised version of the Beck Depression Inventory (BDI-II) was administered at T0. BDI-II is a self-reported, 21-item inventory that measures the severity of depression [27]. Each question is scored from 0 to 3, with higher scores being indicative of more severe depressive symptoms. Based on BDI-II, four categories are identified: Minimal depression (score = 0–13), mild depression (score = 14–19), moderate depression (score = 20–28), and severe depression (score = 29–63) [27]. 

Finally, serum luteinizing hormone (LH), testosterone, sex hormone binding globulin (SHBG), free testosterone (calculated by Vermeulen’s formula), and prolactin levels within the three months preceding the initiation of LIESWT sessions were recorded at T0. 

**Figure 1:**
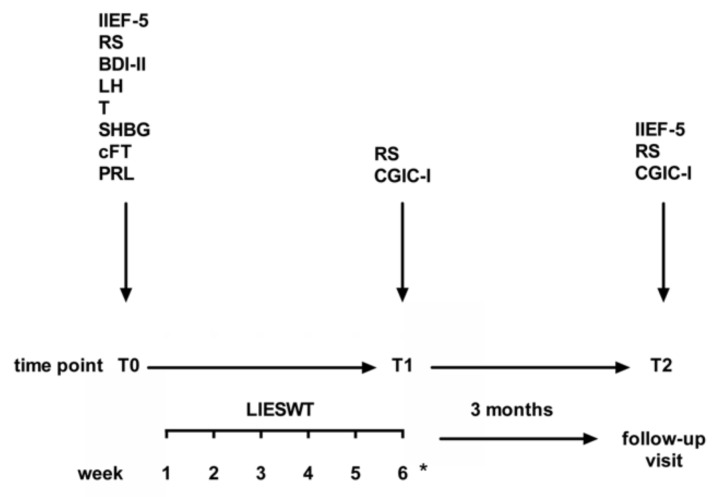
Flow chart of this study. Time points: T0 = first visit prior to commencement of low-intensity extracorporeal shockwaves therapy (LIESWT) sessions (baseline); T1 = last LIESWT session (the sixth for patients who underwent 6 sessions, or the sixth and the twelfth for patients who underwent 12 sessions—see below); T2 = three months after the end of LIESWT sessions. * Four patients, who did not respond or poorly responded to the first LIESWT cycle, underwent another 6 weekly sessions after a 3-week break. Abbreviations: BDI-II = Beck Depression Inventory; cFT = calculated free testosterone; CGIC-I = Clinical Global Impression of Change-Improvement Component; IIEF-5 = International Index of Erectile Function; LH = luteinizing hormone; LIESWT = low-intensity extracorporeal shockwaves therapy; PRL= prolactin; RS = rigidity score; SHBG = sex hormone binding globulin; T = testosterone.

The flow chart of this study is shown in Figure 1.

### Statistical Analysis

Categorical variables were compared by the Fisher’s exact test or the χ^2^-test, as appropriate, whereas continuous variables by the Mann–Whitney test. Correlation between variables was analyzed by bivariate analysis and multistep linear regression. *P* values < 0.05 were considered significant, while *P* values between 0.05 and 0.10 were considered borderline trendwise significant.

## 3. Results

All patients completed the study. Overall, no local or systemic adverse event was recorded. Table 1 summarizes clinical and biochemical characteristics of the 20 patients. Patients were divided into four groups based on the current use of drugs for diabetes, hypertension, hypercholesterolemia, and on past history of myocardial infarction. 

At baseline (T0), the number of PDE5i to which patients failed to respond correlated negatively with RS (*r* = −0.557, *P* = 0.01). After six LIESWT sessions, 16 patients (80%) improved their RS score, but only in 11 patients (55%) erections were hard enough for penetration (RS ≥ 3), compared to none at baseline (*P* = 0.0001). After another six sessions, one more patient out of the four who received these extra sessions reached an RS ≥ 3, thus bringing the total to 12 (60%). Handling RS as a continuous variable also yielded a statistically significant difference (2.5 ± 1.0 at the last session vs. 1.4 ± 0.7 at baseline; *P* = 0.0002). The rate of patients who reported improvement of erections (CGIC-I ≥ 1) was 75% (15/20), but it was 80% (16/20) considering one patient who received 12 LIESWT sessions (Table 2). 

As expected, the three scores used to evaluate erections correlated with each other, as RS at T0 correlated positively with the RS (*r* = 0.474, *P* = 0.03), CGIC-I (*r* = 0.422, *P* = 0.06), and IIEF-5 (*r* = 0.411, *P* = 0.07) at T1, while RS at T1 also correlated with CGIC-I (*r* = 0.659, *P* = 0.002) and IIEF-5 (*r* = 0.506, *P* = 0.04). Finally, CGIC-I correlated positively with IIEF-5 (*r* = 0.539, *P* = 0.01). 

Baseline prolactin levels correlated inversely and trendwisely with the RS at T1 at bivariate analysis (*r* = −0.600, *P* = 0.066), whereas testosterone levels correlated positively with the CGIC-I at multistep linear regression (β = 0.617, *P* = 0.01).

### 3.1. Groups Analysis

Compared to patients aged ≥65 years, those younger than 65 years had higher RS at the end of six sessions (2.7 ± 0.9 vs. 1.8 ± 1.2, *P* = 0.04), and more frequently reached an RS ≥ 3 (9/12, 75% vs. 2/8, 25%, *P* = 0.06). After another six sessions, a 65-year-old patient, out of the four who received these extra sessions, increased his RS from 0 to 3. Therefore, the above-mentioned differences between groups were not confirmed. Overall, age correlated negatively with RS at T1 both at bivariate analysis (*r* = −0.526, *P* = 0.04) and at multistep linear regression (β = −0.95, *t* = −4.957, *P* < 0.0001). These data were confirmed at T2 (see below).

Compared to nondiabetic patients, diabetic patients complained of more severe (IIEF-5 score: 10.8 ± 3.2 vs. 13.9 ± 3.1, *P* = 0.03; IIEF-5 score, erectile domain: 5.0 ± 2.3 vs. 8.1 ± 2.9, *P* = 0.02), less longstanding erectile dysfunction at T0 (5.5 ± 4.8 vs. 10.0 ± 7.3 years, *P* = 0.058). Indeed, the rate of patients with severe to moderate, mild to moderate, and mild erectile dysfunction was 62.5%, 37.5%, and 0% in the diabetes group, while 16.7%, 83.3%, and 0% in the non-diabetes group (*P* = 0.06). The rate of patients who responded to LIESWT (RS at T1 ≥ 3) was similar in the two groups (5/8, 62.5% vs. 7/12, 58.3%). However, only in diabetic patients, those who responded had an IIEF-5 at T0 significantly higher compared with those who did not respond (diabetic patients: 12.5 ± 1.7 vs. 7.3 ± 1.6, *P* = 0.03; nondiabetic patients: 17.3 ± 2.6 vs. 15.0 ± 1.0, *P* = 0.22). Diabetes duration correlated negatively with erectile function domain of the IIEF-5 score (*r* = −0.703, *P* = 0.05). Only in nondiabetic subjects, serum testosterone correlated positively with RS at T1 at the bivariate analysis (*r* = 0.804, *P* = 0.009), while BDI-II score correlated negatively with IIEF-5 score at multistep linear regression (β = −0.447, *t* = 3.573, *P* = 0.04). 

All four patients who had had at least one myocardial infarction suffered from both hypertension and hypercholesterolemia (4/4 vs. 2/16, *P* = 0.003). Duration of hypertension correlated positively with age (*r* = 0.828, *P* = 0.006), and negatively with the RS at T1 (*r* = −0.973, *P* = 0.001). Only in hypertensive patients, impotence duration correlated negatively with calculated free testosterone (*r* = −0.996, *P* = 0.057). Finally, in nonhypertensive patients, BDI-II score correlated negatively with the satisfaction domain of the IIEF-5 score at T1 (*r* = −0.746, *P* = 0.008).

The rate of patients who did not take lipid-lowering drugs and who had T1 RS ≥ 3 was 2.5-fold greater compared to those who took these class of drugs (9/11, 81.8% vs. 3/9, 33.3%, *P* = 0.06) (Table 2). This difference also held upon handling RS as a continuous variable (2.8 ± 0.8 vs. 2.1 ± 1.2, *P* = 0.06). These two groups also differed for the number of cardiovascular comorbidities associated (0.7 ± 0.6 vs. 2.4 ± 1.0, *P* = 0.0009). 

Interestingly, BDI-II was significantly greater in patients with at least one among diabetes, hypertension, hypercholesterolemia, and past history of myocardial infarction compared to those with none of them (6.8 ± 4.6 vs. 1.7 ± 2.2, *P* = 0.03).

### 3.2. Follow-Up

Three months after the end of LIESWT (T2), RS was unchanged compared to T1 (2.4 ± 1.0 vs. 2.5 ± 1.0), and so was the rate of patients who reached erections sufficiently firm for penetration (55% vs. 60%). Also, patients ≥65 years and those with hypercholesterolemia were less likely to report a positive response to LIESWT, with lower RS compared to the corresponding group (≥65 years, 1.6 ± 1.1, vs. <65 years, 2.7 ± 1.0 *P* = 0.056; hypercholesterolemia, 1.8 ± 1.0, vs. nonhypercholesterolemia, 2.7 ± 0.8 *P* = 0.04).

Worthy of note, 60% of patients reported a better response to PDE5i after LIESWT. However, three patients who had reported an improvement of erections (CGIC-I ≥ 1) at T1, defined their erections as “unchanged” at T2 (CGIC-I = 0). Hence, the rate of patients with CGIC-I score ≥1 was 65% (vs. 80% at T1) (Table 2). 

Compared with baseline, IIEF-5 score at T2 was significantly higher (14.4 ± 2.4 vs. 11.6 ± 3.6, *P* = 0.04), owing to improvement of the erectile function domain (8.4 ± 2.1 vs. 5.9 ± 3.0, *P* = 0.03). Indeed, intercourse satisfaction domain also improved, although not significantly (6.0 ± 0.9 vs. 5.7 ± 1.3, *P* = 0.59) (Table 2).

At T2, RS correlated negatively and significantly with age (*r* = −0.497, *P* = 0.04), with BDI-II score (*r* = −0.4692, *P* = 0.049), and with duration of hypertension (*r* = −0.926, *P* = 0.0009), but trendwisely with baseline prolactin levels (*r* = −0.5845, *P* = 0.06). 

## 4. Discussion

In the last decade, several studies have shown the beneficial effects of LIESWT for the treatment of PDE5i-resistant erectile dysfunction [28]. Long-term duration of these effects is still debated [29,30,31,32]. Two are the proposed mechanisms whereby LIESWT improve erectile function: Shear stress and endothelium disruption by growth and implosion of cavitation bubbles in the vessels, which result in neoangiogenesis and endothelial and neuronal nitric oxide synthesis [11]. In the murine model of erectile dysfunction, LIESWT are capable of regenerating penile tissue by mesenchymal stem cells recruitment and regeneration of nerves (via Schwann cells activation), and vessels, with the consequent release of pro-angiogenetic growth factors. In addition, LIESWT also improves erectile function via nitric oxide/cGMP-nondependent mechanisms [33,34,35,36]. 

Our study confirms the beneficial, sustained effect of LIESWT in restoring erections sufficient for penetration in PDE5i-resistant patients. Indeed, three-fifths of patients already regained a regular sexual activity at the end of LIESWT sessions, and this effect was maintained three months after the end of this treatment in all but one patient. These data are in line with those reported at one month and three months of follow-up in four published studies [10,23,28,37]. Furthermore, consistently with Tsai [23], we noticed that LIESWT converted 6/10 PDE5i nonresponders to responders. 

In the literature, the response rate to LIESWT, namely restoration of erections sufficiently firm for penetration, and duration of follow-up ranged widely from 3.5% to 77.5% and from 1 to 12 months [11]. Our 2.8-point improvement over baseline in the total IIEF-5 score at three months after the end of LIESWT, together with the 2.5-point improvement in the IIEF-5 erectile function domain score, matched the changes found by Chung (+2.5) [38] and Fojecki (+2.2) [39], even though they used different devices and different protocols. 

Concerning factors affecting the outcome of LIESWT, a single-arm study on 56 patients demonstrated that age ≥65 years and the presence of ≥3 comorbidities predicted a negative response (RS < 2) to LIESWT [21]. Comorbidities included hypertension, diabetes, ischemic heart disease, and hyperlipidemia [21]. In contrast with this study, another two uncontrolled studies found no difference in either hypertension or dyslipidemia rate according to response to LIESWT [22,23]. In agreement with one study [21], we found that patients aged ≥65 years and those on lipid-lowering drugs were less likely to achieve erections firm enough for penetration after LIESWT. In addition, we found that age correlated inversely with RS both at bivariate and at multistep linear regression. In light of Hisasue’s findings [21], it is not surprising that our patients with history of hypercholesterolemia had more cardiovascular comorbidities. It was reported that atherosclerosis and the ensuing endothelial dysfunction underpin erectile dysfunction [40], and that statins may improve the endothelial function. However, these drugs may also induce erectile dysfunction by reducing testosterone synthesis via 3-hydroxy-3-methylglutaryl-coenzyme A inhibition [40]. As underlined by Kalka, safety and virtual lack of contraindications make LIESWT an ideal, causative treatment for patients with atherosclerosis-associated erectile dysfunction [41]. 

Not surprisingly, we showed that, compared to nondiabetic patients, diabetic patients had a baseline erectile dysfunction that was more severe (−3.1 IIEF-5 erectile function score) and less longstanding (−4.5 years). Also, diabetes duration correlated inversely with the erectile function domain of IIEF-5 score. Because of penile microangiopathy and neuropathy, the prevalence of impotence is three-fold greater in diabetic patients [8,35,42], in whom it can appear 10–15 years earlier than in nondiabetic patients [43]. In our cohort, the eight diabetic patients had HbA1c levels not adequately controlled (mean 8.2%). In diabetic mice, LIESWT has been demonstrated to improve erections with effectiveness comparable to sildenafil [33,35,44,45]. Combination of LIESWT and sildenafil acts synergistically on the erectile dysfunction [35]. In humans, Reisman reported greater effectiveness of LIESWT in nondiabetics vs. diabetic patients (88.2% vs. 70.8%) [46]. Similarly, Bechara recently reported that only 3/24 (12.5%) responders were diabetics, in contrast with 7/16 nonresponders (43.7%, *P* = 0.06) [22]. In our cohort, although the response rate was similar in diabetic patients compared to nondiabetic patients, only within the former group, responders had a higher baseline IIEF-5 compared to nonresponders, namely severe erectile dysfunction in diabetic patients was less likely to improve after LIESWT. Relative resistance to LIESWT by diabetics may result from the underlying endothelial dysfunction with consequent altered NO synthesis as well as from the impaired recruitment of mesenchymal stem cells [47]. 

In addition, Reisman et al. showed that the duration of erectile dysfunction is correlated negatively with the treatment success. In particular, patients who experienced erectile dysfunction for at least 10 years, had a lower IIEF score compared to those with a shorter history of erectile dysfunction [46]. In our cohort, age but not impotence duration negatively influenced the IIEF-5 score at the third month of follow-up.

There are four main limitations of the present study: (i) The relatively small sample size, due to selection criteria and the cost of LIESWT, which was totally covered by the patients; (ii) the short follow-up, for which no conclusion can be drawn on the long-term effects of LIESWT; (iii) the absence of an arm sham-treated, even though sham treatment can being performed in different ways [11]; (iv) the lack of hormone levels measurement and BDI-II assessment also at T1 and T2. Long term, sham-controlled, multicentric studies might overcome these limitations.

In conclusion, LIESWT is a safe and effective treatment for patients with vasculogenic erectile dysfunction who do not respond or respond poorly to PDE5i. The current international guidelines do not include LIESWT as a treatment option for erectile dysfunction. The European Academy of Urology guidelines state that clear recommendations on the use of LIESWT cannot be given since current data are limited [48]. We have demonstrated that age ≥65 years, diabetes, and hypercholesterolemia influence early and negatively the outcome of LIESWT. Nevertheless, pituitary-thyroid and pituitary-gonad axes seem to play a marginal role in response to this treatment, as we enrolled euthyroid, eugonadal, and normoprolactinemic patients. Further studies on certain populations, such as patients treated for hypogonadism, hyper/hypothyroidism, and hyperprolactinemia, are warranted.

## Figures and Tables

**Table 1:** Baseline characteristics of the 20 patients.

Parameter	
Age (m ± SD)	58.5 ± 10.3 years
Diabetes (number of patients (%), duration (m ± SD), HbA1c (m ± SD))	8 (40%), 13.9 ± 9.7 years, 8.2 ± 2.0%
Hypertension (number of patients (%), duration (m ± SD))	9 (45%), 5.7 ± 6.1 years
History of MI (number of patients (%))	4 (20%)
Hypercholesterolemia (number of patients (%), duration (m ± SD))	9 (45%), 8.4 ± 6.1 years
ED duration (m ± SD)	8.2 ± 6.7 years
Number of PDE5i to which patients failed to respond (m ± SD) PDE5i (number of patients)	1.9 ± 0.9 avanafil (3), sildenafil (12), tadalafil (19), vardenafil (5)
i.c. Alprostadil (number of patients (%))	2 (10%)
LH (mU/mL) (m ± SD)	3.8 ± 1.3
Total testosterone (nmol/L) (m ± SD) ^	15.9 ± 6.2
SHBG (nmol/L)	38.2 ± 11.2
Calculated free testosterone (pmol/L) (m ± SD)	330.7 ± 95.0
Prolactin (μU/mL) (m ± SD)	191.5 ± 85.1
Rigidity Score (m ± SD) Erection hardness #	1.4 ± 0.7 0:2 (10%) 1:8 (40%) 2:10 (50%) 3:0 4:0
IIEF-5 (m ± SD) ED severity* (number of patients (%))	11.6 ± 3.6 severe: 4 (20%) moderate: 3 (15%) mild to moderate: 13 (65%) mild: 0
IIEF-5 EFD (m ± SD)	5.9 ± 3.0
IIEF-5 ISD (m ± SD)	5.7 ± 1.3
BDI-II Depression severity ** (number of patients (%))	5.8 ± 4.7 minimal: 19 (95%) mild: 1 (5%) moderate: 0 severe: 0

Abbreviations: ED = erectile dysfunction; EFD = erectile function domain; i.c. = intracavernosal; IIEF-5 = International Index of Erectile Function; ISD = intercourse satisfaction domain; MI = myocardial infarction. ^ Two diabetic patients had total testosterone levels <11 mmol/L, whereas free testosterone was >220 pmol/L because of low-normal levels of SHBG. In these patients, low total testosterone was not confirmed at a subsequent measurement during the follow-up. Therefore, we did not rule exclude them in the final analysis. # Rigidity Score: No erection (0), larger penis but not hard (1), hard penis but not enough for penetration (2), penis hard enough for penetration but not completely hard (3), penis completely hard and fully rigid (4). * IIEF-5 score: Severe ED (1–7), moderate ED (8–11), mild to moderate ED (12–16), and mild ED (17–21). ** BDI-II: Minimal depression (0–13), mild depression (14–19), moderate depression (20–28), and severe depression (29–63).

**Table 2:** Rigidity score (RS) and Clinical Global Impression of Change (CGIC-I) at baseline (T0), at the last LIESWT session (the sixth for patients who underwent six sessions, or the sixth and the twelfth for patients who underwent 12 sessions) (T1), and three months after the end of LIESWT sessions (T2) based on history of diabetes, hypertension, hypercholesterolemia, and myocardial infarction.

	*N*	T0	T1		T2	
		RS ≥ 3	RS ≥3	CGIC-I ≥ 1	RS ≥ 3	CGIC-I ≥ 1
**All**	20	0 ***,****	12 (60%) ****	16 (80%)	11 (55%) ***	13 (65%)
**Diabetes, yes**	8	0 *	5 (62.5%) *	5 (62.5%)	5 (62.5%) *	3 (37.5%)
**Diabetes, no**	12	0 *, **	7 (58.3%) **	11 (91.7%)	6 (50%) *	10 (83.3%)
**Hypertension, yes**	9	0 *, **	6 (66.7%) **	8 (88.9%)	5 (55.5%) *	6 (66.7%)
**Hypertension, no**	11	0 *	6 (54.5%) *	8 (72.7%)	6 (54.5%) *	7 (63.6%)
**Myocardial infarction, yes**	4	0	2 (50%)	3 (75%)	2 (50%)	2 (50%)
**Myocardial infarction, no**	16	0 ***	10 (62.5%) ***	13 (81.2%)	9 (56.2%) ***	11 (68.7%)
**Hypercholesterolemia, yes**	9	0	3 (33.3%) ^	7 (77.8%)	3 (33.3%)	6 (66.7%)
**Hypercholesterolemia, no**	11	0 **,***	9 (81.8%) ***^	9 (81.8%)	8 (72.7%) **	7 (63.6%)

Comparison with T0: * 0.01 ≤ *P* < 0.05; ** 0.001 ≤ *P* < 0.01; *** 0.0001 ≤ *P* < 0.001; **** *P* < 0.0001. Comparison with the corresponding group: ^ 0.05 ≤ *P* < 0.10.
